# Catatonia and Opioid Withdrawal: A Case Report

**DOI:** 10.7759/cureus.56396

**Published:** 2024-03-18

**Authors:** Synthia Lay, Long L Nguyen, Arul Sangani

**Affiliations:** 1 Psychiatry, Kaweah Delta Health Care District, Visalia, USA

**Keywords:** bush-francis catatonia scale, benzodiazepine challenge, illicitly manufactured fentanyl, opioid use, retarded catatonia, geriatric psychiatry, opioid withdrawal

## Abstract

In this case report, we present an 82-year-old female who was diagnosed with catatonia after she exhibited immobility, mutism, withdrawal, and stereotypy during a hospitalization for altered mental status. Fentanyl was found in her urine toxicology, and it was later discovered that she had been taking non-prescription pills from Mexico that were likely the source of the fentanyl. Her catatonia quickly remitted with benzodiazepine treatment. This case underscores previously unknown risks of substance use, which has grown especially important to psychiatric care considering how rampant the opioid epidemic has become. More so, these risks extend beyond opioid use disorders since other non-prescription drugs are commonly laced with fentanyl. Not only does this education need to be given to providers and patients alike, but further research should be conducted to establish and quantify the risk of catatonia with opioid withdrawal.

## Introduction

Catatonia is a neuropsychiatric syndrome characterized by motor, vocal, affective, and behavioral abnormalities such as stupor, catalepsy, waxy flexibility, mutism, negativism, posturing, mannerisms, stereotypy, agitation, grimacing, echolalia, and echopraxia [1]. It is often associated with schizophrenia and mood disorders but is also recognized as a sequela of various medical conditions [2]. It is important to note that catatonia is not conceptualized as a separate diagnostic entity, but rather a constellation of symptoms due to an underlying disorder [2]. One study identified the underlying causes of 148 patients with catatonia and found a distribution as follows: 46% due to bipolar disorder or unipolar depression, 26% due to psychotic disorder, 20% due to general medical condition, and 8% due to another psychiatric disorder [3]. Mainstay treatment consists of benzodiazepines or electroconvulsive therapy (ECT) in addition to treating the underlying condition [2]. Recognition of catatonia is critical, and treatment should be prompt because if left untreated, this syndrome can lead to malnourishment, deep vein thrombosis, pulmonary embolism, and aspiration amongst other severe or life-threatening consequences [4].

While causes of catatonia vary, there has been increasing evidence linking catatonia to substance use [5]. The literature is limited with few studies identifying a relationship between catatonia and withdrawal from alcohol, opioids, and sedative-hypnotics [5]. Only a handful of case reports have discussed the possible connection between catatonia and opioid use. Hypotheses postulate that opioids can induce catatonic symptoms by altering neurotransmitter metabolism in different parts of the brain, particularly the basal ganglia [6]. Here, we describe the case of an 82-year-old female who developed catatonia from opioid withdrawal and was successfully treated with benzodiazepines.

## Case presentation

An 82-year-old woman with a history of depression, undiagnosed dementia, hypertension, hyperlipidemia, chronic kidney disease stage IIIa, polymyalgia rheumatica, and coronary artery disease presented to the hospital with altered mental status for one day in the setting of a recently treated urinary tract infection. In the emergency department, laboratory analysis was significant for mild leukocytosis, mildly elevated creatinine, and elevated troponin levels. Urinalysis, CT of the head scan, and electrocardiogram were normal. She was given a 1 L bolus of lactated Ringer's solution, and her mentation improved. After her troponin levels were observed to be downtrending, she was admitted to the medical floor for observation, and her laboratory values improved the following day. She returned to her baseline two days after admission and was about to be discharged until she became unresponsive. She was unable to move, speak, or follow commands. A rapid response team was called and tried to drop her hand on her head, but she withdrew before her hand could land on her head. Her vital signs were within normal limits except for elevated blood pressure.

Neurology was consulted for further evaluation. History collected from the patient's family revealed that the patient had been taking Artri King pills for back pain for a couple of weeks. The patient's family purchased these pills from Mexico and did not know their contents, but they were marketed as "all natural." On examination, the patient was awake, but she was minimally reacting to her environment as she did not divert her attention to any stimuli or respond to any questioning. She did not move her head or extremities and resisted passive movement. No focal neurological deficits were appreciated. Laboratory results were largely unremarkable, including complete blood count, complete metabolic panel, thyroid function panel, B12/folate levels, HIV screen, rapid plasma reagin (RPR) screen, and COVID antibodies. Notably, the patient's urine toxicology was positive for fentanyl. Electroencephalogram was normal. It was suspected that the Artri King pills were likely the source of fentanyl found in the urine as the patient did not have any history of substance use. According to the FDA, Artri King pills contain hidden drug ingredients, including, but not limited to, dexamethasone and diclofenac [7]. Given the patient's exam findings and normal laboratory and imaging results, she was recommended a psychiatry consult for suspected catatonia.

Upon psychiatric evaluation, the patient did not speak or make eye contact with the interviewer. She stared for several seconds at a time and repeatedly used her left hand to scratch the back of her head. She did not withdraw to pain, and it was confirmed that she had not been eating or drinking for two days. She scored a 13 on the Bush-Francis Catatonia Rating Scale (BFCRS) [2] for immobility, mutism, staring, stereotypy, and withdrawal. A lorazepam challenge was done with one dose of intravenous lorazepam 1 mg. Approximately 30 minutes later, the patient was alert and able to engage in conversation, dropping her Bush-Francis score to a 4 and confirming the diagnosis of catatonia. She began treatment with scheduled intravenous lorazepam 1 mg three times a day.

The patient's catatonia significantly improved the following day. She was able to fully participate in the interview and remained with only minimal hypoactivity. She tolerated the treatment well and denied having any adverse effects of lorazepam. The next day, the patient had no symptoms of catatonia, scoring a 0 on the BFCRS. Given the excellent response to benzodiazepine treatment, her intravenous lorazepam was transitioned to oral lorazepam 2 mg three times a day. After three days, the lorazepam dose was decreased to 1 mg three times a day. The patient was subsequently discharged to a skilled nursing facility with instructions to follow up with a psychiatrist to gradually taper off the lorazepam.

## Discussion

According to the Diagnostic and Statistical Manual of Mental Disorders, Fifth Edition (DSM-5), catatonia is defined as the presence of at least three of the following: catalepsy, waxy flexibility, stupor, agitation, mutism, negativism, posturing, mannerisms, stereotypies, grimacing, echolalia, and echopraxia [1]. In the case above, our patient met the criteria for catatonia as she demonstrated stupor, mutism, and stereotypy and responded positively to the lorazepam challenge. The etiology of catatonia was most likely withdrawal from fentanyl given that the patient did not have any other predisposing psychiatric condition and developed symptoms after discontinuation of the fentanyl-containing substance. However, it is plausible that the Artri King pills contained another ingredient that could have contributed to the catatonia. The patient's age and neurocognitive impairment likely put her at greater risk of developing complications from substance use. Although the patient had a remote history of depression according to her medical record, her family denied any ongoing depression.

The literature on opioid-induced catatonia is sparse. Multiple anesthesia case reports have commented on post-operative catatonia and rigidity relieved with naloxone following the administration of opioids [8,9]. Meanwhile, very few case reports have established opioid withdrawal as a potential causative factor of catatonia [10]. Other studies have attempted to explain the role of the endogenous opioid system in producing the muscular rigidity and akinesia seen in catatonia [6]. To some extent, opioids decrease both excitatory (glutaminergic) and inhibitory (gamma-aminobutyric acid (GABA)) neurotransmission [11]. Because catatonia has been postulated to be caused by excess glutamate and reduced GABA activity [2], opioid use can potentially lead to catatonia via GABA hypoactivity. Alternatively, opioid withdrawal may also contribute to catatonia via rebound glutamate hyperactivity.

To our knowledge, this case report is the only one of its kind to focus on catatonia precipitated by opioid withdrawal without the influence of other substances. There appears to be more evidence of catatonia induced by benzodiazepine withdrawal, but less is known about the risks of rare psychiatric complications from opioid withdrawal [12]. Another interesting aspect about this case is the source of opioids being found in supplements distributed from Mexico, which should remind providers to inquire about non-prescription pill usage during history taking.

## Conclusions

Catatonia can be a rare consequence of opioid use. Prompt recognition of catatonic symptoms and treatment with benzodiazepines led to the resolution of our patient's catatonia. This case highlights the importance of investigating the use of unknown substances and supplements given the rising growth and spread of new drugs. Not only should future studies further examine the role of opioids in contributing to catatonia, but they should additionally research strategies to combat addiction and the opioid epidemic.
